# Utilization rates of intravenous thrombolysis for acute ischemic stroke in Asian countries:: A systematic review and meta-analysis

**DOI:** 10.1097/MD.0000000000035560

**Published:** 2023-10-20

**Authors:** Bikram Prasad Gajurel, Gaurav Nepal, Vikash Jaiswal, Song Peng Ang, Priyanshu Nain, Nishat Shama, F.N.U. Ruchika, Sujan Bohara, Sanjeev Kharel, Jayant Kumar Yadav, Jillian Reeze T. Medina, Abhigan Babu Shrestha

**Affiliations:** a Department of Neurology, Tribhuvan University Institute of Medicine, Maharajgunj, Kathmandu, Nepal; b Department of General Medicine, Rani Primary Healthcare Centre, Biratnagar, Nepal; c Larkin Community Hospital, South Mimai; d Division of Internal Medicine, Rutgers Health/Community Medical Center, NJ; e Department of Medicine, Maulana Azad Medical College, New Delhi, India; f Bangladesh Institute of Research and Rehabilitation in Diabetes, Endocrine and Metabolic Disorders, Dhaka, Bangladesh; g Department of Surgery, JJM Medical College, Davangere, India; h Department of Internal Medicine, Nepalese Army Institute of Health Science, Kathmandu, Nepal; i Department of Internal Medicine, Maharajgunj Medical Campus, Tribhuvan University Institute of Medicine, Maharajgunj, Kathmandu, Nepal; j Department of Neurology, Tufts Medical Centre, Boston; k Manila Central University - Filemon D. Tanchoco Medical Foundation College of Medicine, Philippines; l Department of Internal Medicine, M Abdur Rahim Medical College, Dinajpur, Bangladesh.

**Keywords:** Asia, ischemic stroke, stroke, thrombolysis, tPA

## Abstract

**Background:**

Despite intravenous thrombolysis (IVT) being used for the treatment of acute ischemic stroke (AIS) for over two decades, its accessibility remains limited in various regions of the world. The Asian region, which experiences the highest age-standardized incidence of AIS, currently lacks comprehensive data on the utilization of IVT.

**Aims:**

This study aimed to provide precise estimates of IVT usage for AIS in Asian countries.

**Methods:**

A literature search was conducted on PubMed and Google using appropriate search terms. English language, peer reviewed articles published after 2010 were included in the analysis. The pooled proportion was calculated utilizing the DerSimonian and Laird random-effects model. Additionally, a subgroup analysis was conducted, taking into account factors such as the study's country, the economic status of the country, specific Asian regions, publication year (before 2015 and from 2015 onwards), study location, study setting, hospital stroke protocol, and national stroke guidelines.

**Results:**

67 observational studies with 778,046 patients with AIS were included in the meta-analysis. The overall utilization rate of IVT was found to be 9.1%. High-income countries had a higher rate (11.3%) compared to lower-middle-income (8.1%) and upper-middle-income countries (9%). Central and North Asia had the highest rate (17.5%) and Southeast Asia had the lowest rate (6.8%). Studies conducted after 2015 had a higher thrombolysis rate (11.3%) compared to those before 2015 (1.5%). Presence of hospital stroke protocols (10.7%) and national stroke guidelines (10.1%) were associated with higher thrombolysis rates.

**Conclusion:**

The overall utilization rate of IVT for AIS in Asia stood at 9.1%, showcasing noteworthy disparities across countries, regions, and income brackets. To improve thrombolysis rates in the region, addressing prehospital delays, increasing public awareness, and implementing stroke protocols and national guidelines are key strategies.

## 1. Introduction

**Figure 1.**
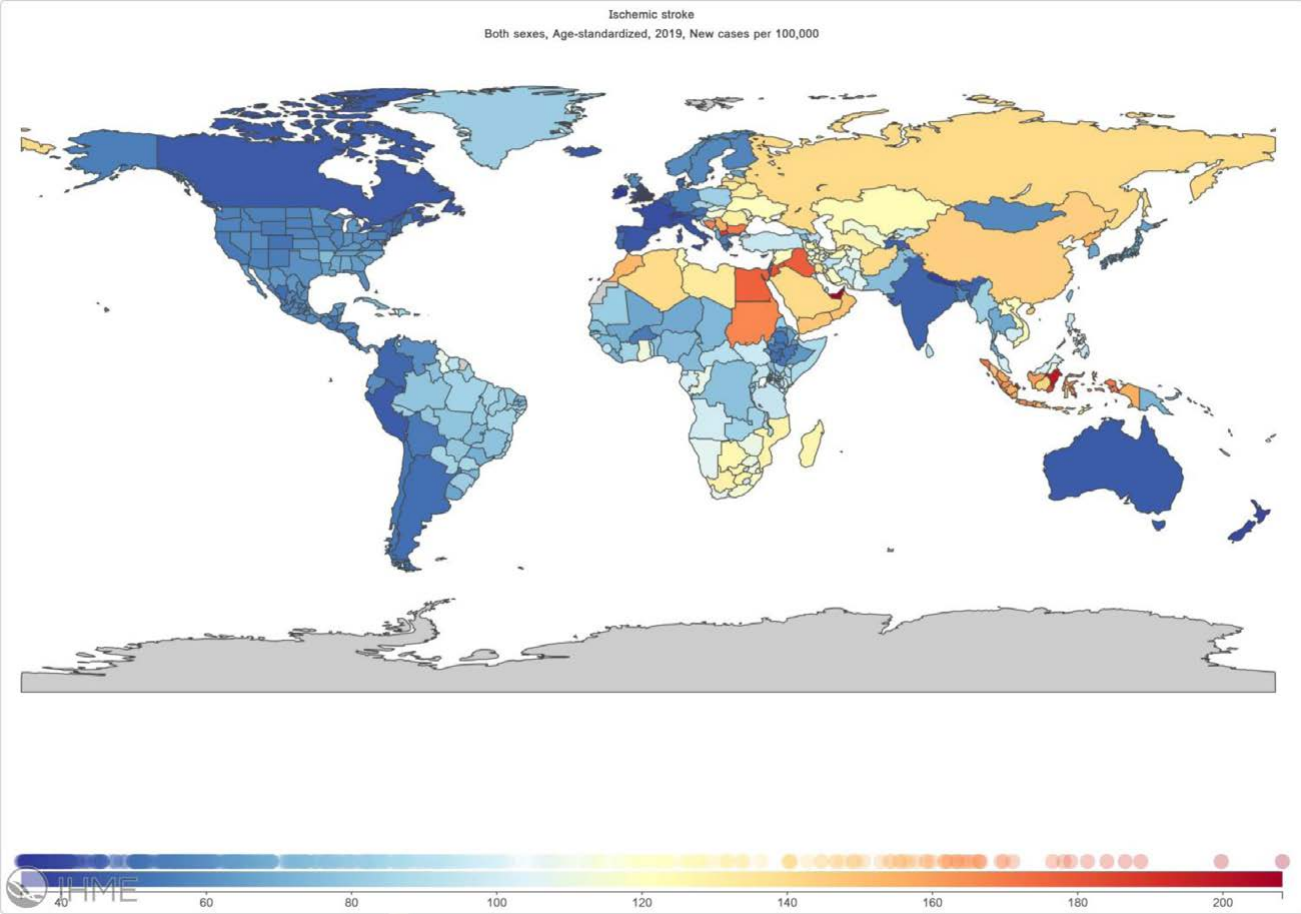
Institute for health metrics and evaluation’s visualization of GBD 19 data showing the age-standardized incidence of ischemic stroke in the Asian region. GBD = global burden of disease.

The global burden of disease study shave shown that in 2019 there were 12.2 million stroke incidents, 101 million prevalent strokes, 143 million disability-adjusted life years lost to stroke, and 6.55 million stroke fatalities. Stroke remained the second largest cause of death globally in 2019 and the third major cause of death and disability when combined. Between 1990 and 2019, the absolute number of incident strokes climbed by 70%, prevalent strokes by 85%, stroke fatalities by 43%, and disability-adjusted life years attributable to stroke increased by 32%. Ischemic stroke accounted for 62% of all strokes reported in 2019; According to the Institute for Health Metrics and Evaluation’s visualization of global burden of disease 19 data, the age-standardized incidence of ischemic stroke is considerably greater in the Asian region (Fig. 1).^[1,2]^

At present, the only evidence-based standard treatments for patients experiencing acute ischemic stroke (AIS) are recanalization therapies. The 2 primary methods are intravenous thrombolysis (IVT) and mechanical thrombectomy. IVT involves the administration of a clot-dissolving medication, typically alteplase, through an intravenous infusion to dissolve the clot causing the ischemic stroke. Mechanical thrombectomy, on the other hand, involves physically removing the clot using specialized devices inserted into the blocked blood vessel.^[3]^ IVT with alteplase is currently the most widely available, affordable, and commonly used therapy for AIS. It is recommended for individuals who can receive treatment within 4.5 hours of stroke onset. However, despite being in practice for over 2 decades, the coverage of IVT remains inadequate globally.^[4]^ Astonishingly, only about 30% of countries worldwide have reported performing IVT for AIS. Moreover, there is significant disparity in IVT utilization among different nations, with rates ranging from 10% to 15% in high-income countries to <2% in low- and middle-income countries.^[5]^

Of particular concern is the Asian region, where the age-standardized incidence of ischemic stroke is considerably higher compared to other regions.^[6]^ Unfortunately, there is currently no specific data on IVT utilization in Asia, which makes it challenging to understand the extent of access to this crucial treatment in the region most affected by ischemic stroke. This study aims to provide accurate, region-specific estimates of IVT usage for AIS in Asia.

## 2. Methodology

This systematic review and meta-analysis is reported in accordance with the Preferred Reporting Items for Systematic Reviews and Meta-Analyses checklist and flow diagram for manuscript format development as guideline.^[7]^ Ethical approval was not required as this study is a systematic review and meta-analysis.

### 2.1. Eligibility criteria

For inclusion, the studies had to fulfill the following criteria: Original, peer-reviewed, observational research articles from Asian region published in English; Studies included adult patients with AIS of anterior or posterior circulation; Patients with AIS received IVT (alteplase or tenecteplase or urokinase); Studies should report the overall rate of intravenous thrombolysis among AIS patients. The exclusion criteria were: AIS studies from Asia that fail to present data on thrombolysis rate; Studies reporting rate of mechanical thrombectomy; Case reports, series and review articles; Clinical trials were also excluded as they do not showcase the real-world rate of utilization of thrombolysis.

### 2.2. Search strategy

Our search databases included PubMed and Google, the latter knowing that all Asian studies might not have been published in PubMed-indexed journals. Our search was supplemented by scanning of reference lists of relevant systematic reviews. We excluded randomized controlled trials as they do not actually show the real-world utilization rate of thrombolysis. We did not consider gray literature due to the desire to focus on peer-reviewed studies. We only searched studies published after 2010 as it depicts more accurate result because IVT were lately introduced in Asia except in developed countries like Japan, Hong Kong, South Korea and Singapore. Our final search strategy for PubMed was: (((“Ischemic Stroke”[MeSH Terms] OR “Cerebral Infarction” OR “Brain Infarction”) AND (“Thrombolytic Therapy”[Mesh] OR “Tissue Plasminogen Activator”[Mesh] OR “alteplase” OR “urokinase” OR “Tenecteplase” OR “thrombolysis” OR “rt-PA”)) AND (“cross-sectional study” OR “prevalence” OR “rate”)) AND (“Afghanistan” OR “Armenia” OR “Azerbaijan” OR “Bahrain” OR “Bangladesh” OR “Bhutan” OR “Brunei” OR “Myanmar” OR “Cambodia” OR “China” OR “Egypt” OR “Georgia” OR “Hong Kong” OR “India” OR “Indonesia” OR “Iran” OR “Iraq” OR “Israel” OR “Japan” OR “Jordan” OR “Kazakhstan” OR “North Korea” OR “South Korea” OR “Kuwait” OR “Kyrgyzstan” OR “Laos” OR “Lebanon” OR “Macau” OR “Malaysia” OR “Maldives” OR “Mongolia” OR “Nepal” OR “Oman” OR “Pakistan” OR “Philippines” OR “Qatar” OR “Russia” OR “Saudi Arabia” OR “Singapore” OR “Sri Lanka” OR “Syria” OR “Taiwan” OR “Tajikistan” OR “Thailand” OR “Timor-Leste” OR “Turkmenistan” OR “Turkey” OR “United Arab Emirates” OR “Uzbekistan” OR “Vietnam” OR “Yemen”). For Google, our search was flexible, and consisted of random combination of “Ischemic stroke,” “thrombolytic therapy,” “thrombolysis” combined with name of each Asian country. Full strategy is given in Supplemental Digital Content 1, http://links.lww.com/MD/K249.

### 2.3. Study selection

The shortlisted studies were imported into the Mendeley library, and duplicates were removed. Following that, a manual check was performed to remove any remaining duplicates. Reviewers were un-blinded to author and institution details of citations. To maximize consistency in assessing inclusion and exclusion criteria among all reviewers, we did calibration pilot exercises for title and abstract screening and online group videoconferencing. This training emphasized the need for sensitivity in citation selection in the title and abstract phase. Independently and in duplicate, reviewers screened titles and abstracts for potentially relevant studies. Because of the anticipated very large number of potentially relevant citations, the agreement of 2 members of the review team was required for citation selection at the title and abstract phase; disagreements was adjudicated by a third reviewer. Papers were initially reviewed independently by 5 reviewers (GN, SB, SK, VJ, JM) based on title, keywords, and abstract. Articles that passed the initial screening were then thoroughly reviewed by the same reviewers. Based on authorship, hospital setting, and recruitment period, population overlap was evaluated. In cases of overlap, studies of higher quality or those which consisted of larger sample sizes were included.

### 2.4. Data extraction

#### 2.4.1. Reviewers and data extraction template

Individual reviewers were responsible for extracting data from the selected articles. They used Microsoft Excel as the data extraction tool. The data extraction template in Excel was designed based on the specific variables of interest that were relevant to the study’s objectives.

#### 2.4.2. Pilot testing and group discussion

Before proceeding with the full data extraction, the data sheet underwent multiple iterations. To ensure consistency and reliability in the data extraction process, pilot testing was conducted on a subset of selected articles. The review group members engaged in group discussions to resolve any ambiguities or issues that arose during the pilot testing.

#### 2.4.3. Review of data entry consistency

To maintain data accuracy and consistency, a review group member reviewed each cluster of data entry. This step was crucial in detecting any discrepancies or errors in the initial data extraction and ensuring the information was entered correctly.

#### 2.4.4. Shared spreadsheet and collaborative extraction

After the pilot testing and data entry consistency review, the final included studies were compiled, and the data extraction was performed collaboratively by 4 authors (JM, RR, PP, and NS). They worked on a shared spreadsheet, enabling real-time updates and facilitating efficient collaboration.

#### 2.4.5. Cross-matching and resolution of discrepancies

Once the data extraction was completed, VJ cross-matched the extracted data to identify any disagreements or inconsistencies. In cases where discrepancies were found, the authors revisited the respective papers to reevaluate the information. If necessary, any unresolved disagreements were addressed with the assistance of GN.

#### 2.4.6. Extracted variables

The following information was extracted from each study:

- First author.
- Study site.
- Year of publication.
- Sample size.
- Mean age of patients in the study.
- Thrombolysis rate: The percentage of patients who received thrombolysis among the study population.
- Thrombolysis site: The medical facility where thrombolysis was administered.
- Thrombolytic agents used: The specific drugs (alteplase/tenecteplase/urokinase) used for thrombolysis.
- Mean onset to door time: The average time between stroke onset and the patient’s arrival at the medical facility (door).
- Mean door to needle time: The average time between a patient’s arrival at the medical facility and the initiation of thrombolysis treatment (needle).
- Causes of prehospital delay: The factors contributing to delays in seeking medical attention from the time of stroke onset.
- Reasons for not using thrombolysis: The factors or reasons cited for not administering thrombolysis to eligible patients.
- Prehospital interventions: Any interventions or actions taken before the patient’s arrival at the medical facility.
- Study setting: The specific type of healthcare setting where the study was conducted
- Use of hospital stroke protocol: Whether the medical facility followed a specific stroke protocol for treatment and management.
- Presence of National Stroke Guideline: Whether there was a national guideline or protocol for stroke manage ment in the country where the study was conducted.

#### 2.4.7. Data verification and missing information

In cases where required data was missing, not reported, or presented in an unusual format in the published papers, the corresponding authors of those papers were contacted via email. This step aimed to seek clarification and obtain the missing or unclear information directly from the original authors. Additionally, supplementary material related to the main articles was investigated to gather further details, if necessary.

### 2.5. Statistical analysis

A meta-analysis of the proportion was performed and expressed as a pooled proportion with a 95% confidence interval (CI) for overall rate of IVT in Asian region. Representative forest plots with 95% CIs showing individual studies and the combined effect size were generated to provide an overview of the results for all outcomes. The DerSimonian and Laird random-effects model was used for meta-analysis as heterogeneity was high. Visual inspection of the funnel plot and Egger test were used to assess publication bias. We also ran a sensitivity analysis, removing 1 study at a time to see how it affected the effect size estimate. The subgroup analysis was conducted on several headings: country of the study, Gross Domestic Product of the country, regions of the Asia, year of publication (before 2015 and 2015 onwards), study site, study setting, hospital stroke protocol and national stroke guideline. Descriptive statistics were used to analyze data other than rate of thrombolysis. Continuous data were reported as mean with standard deviation. Categorical data were expressed as frequency and percentage. A statistically significant *P* value threshold of .05 was used. STATA software Version 16 was employed for meta-analysis (StataCorp). IBM SPSS Version 25 (Armonk, NY: IBM Corp.) was used for descriptive analysis.

## 3. Results

### 3.1. Baseline features of included studies and the patient cohort

**Figure 2.**
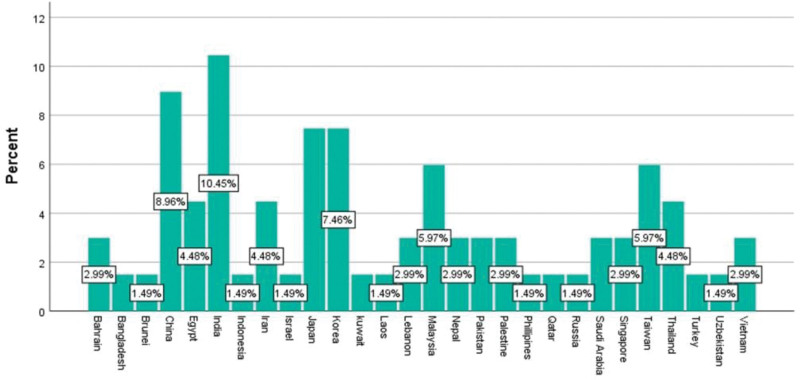
The distribution of included studies based on country of origin.

**Figure 3.**
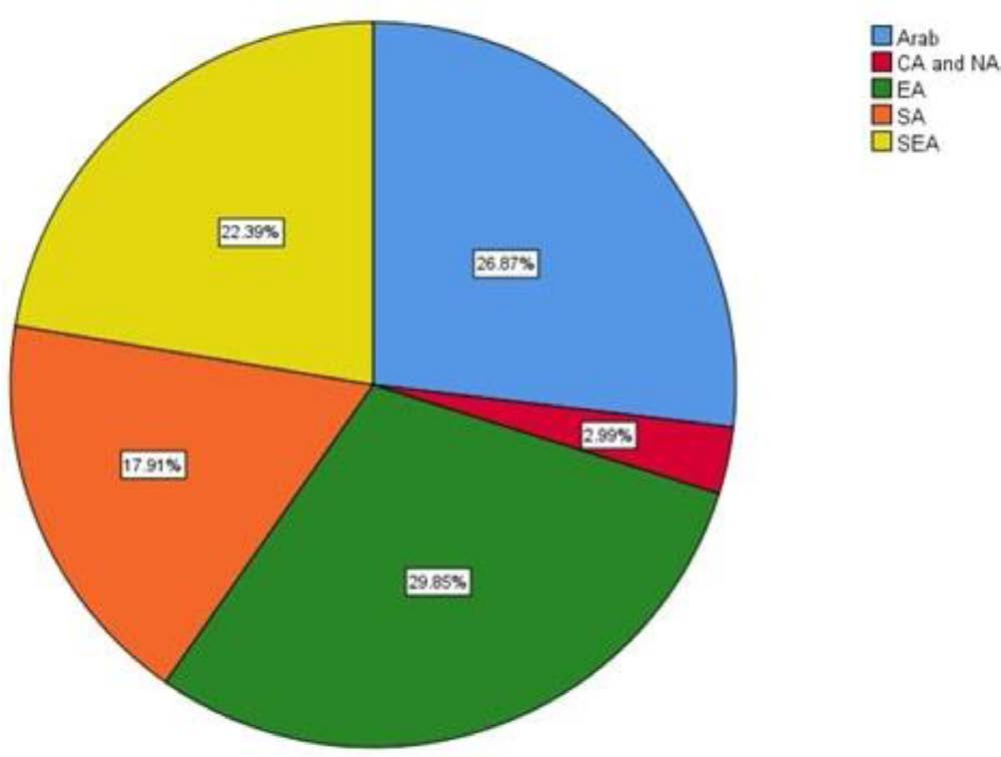
Regional distribution of included studies.

In total, 739 (Pubmed = 259, Google Scholar = 480) articles were identified after a thorough database search. After the exclusion of duplicates and those not meeting inclusion criteria, 67 observational studies (Figure S1, Supplemental Digital Content, http://links.lww.com/MD/K250) with a total of 778,046 patients with ischemic stroke were included in the final review and meta-analysis. All studies were observational studies, the distribution based on country of study is shown in Figure 2. In terms of regional distribution of studies, East Asia was the most common, followed closely by Arab, Southeast Asia, South Asia and lastly Central and North Asia (Fig. 3). In terms of the income level stratified by GDP of these countries, 35.8 % of them were classified as high-income countries, 40.3% were of lower-middle-income countries (LMICs) and the rest (23.9%) being upper-middle-income countries.

**Figure 4.**
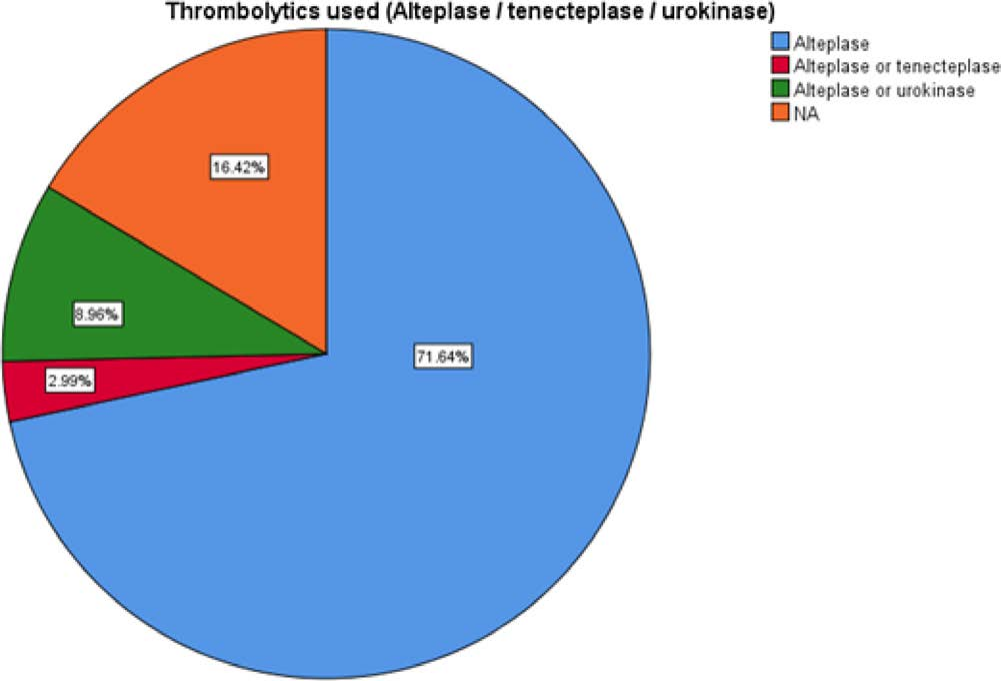
The frequency of various thrombolytics administered.

**Figure 5.**
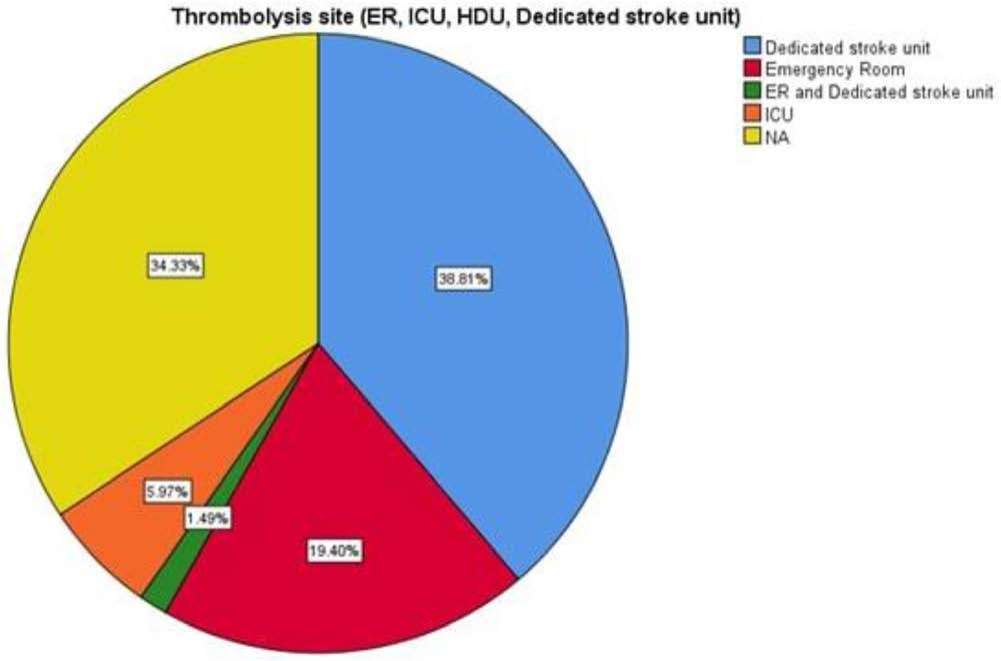
The frequency of various site of thrombolysis.

**Figure 6.**
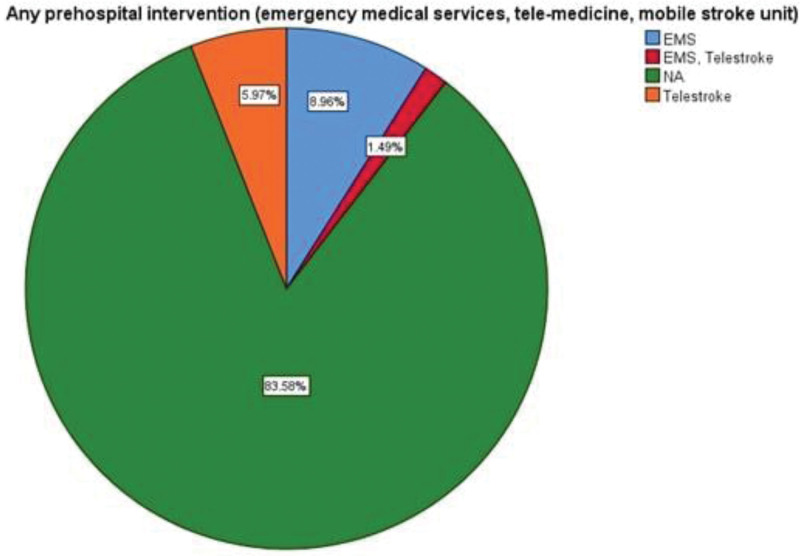
The frequency of EMS and telestroke service utilization. EMS = emergency medical service.

The pooled mean age of ischemic stroke patients was 65.43 ± 9.018 years (34 studies). The pooled mean onset to door time was 236.86 ± 47.32 minutes (25 studies) whereas mean door to needle time was 81.02 ± 16.49 minutes (25 studies). Use of thrombolytics were reported in 56 studies and were described in terms of Alteplase, Alteplase or Urokinase and Alteplase or Tenecteplase. The most common thrombolytics administered was Alteplase (71.8%), followed by Alteplase or Urokinase (9%) and Alteplase or Tenecteplase (3%) (Fig. 4). Among the sites where thrombolysis treatment was being administered, the most common site was a dedicated stroke unit (38.8%), followed by emergency room (19.4%) and ICU (6%) (Fig. 5). Prehospital interventions were described in terms of emergency medical service (EMS), telestroke or a combination of EMS and telestroke. Among the reported 11 studies, EMS was used in 6 studies, telestroke in 4 studies and both EMS and telestroke were employed in 1 study (Fig. 6). In our study, we observed the most important reason for not using thrombolysis to be a prehospital delay due to transportation issues, failure to identify signs of stroke, lack of public awareness, lack of emergency medical system, mobile stroke unit and telemedicine practices.

**Figure 7.**
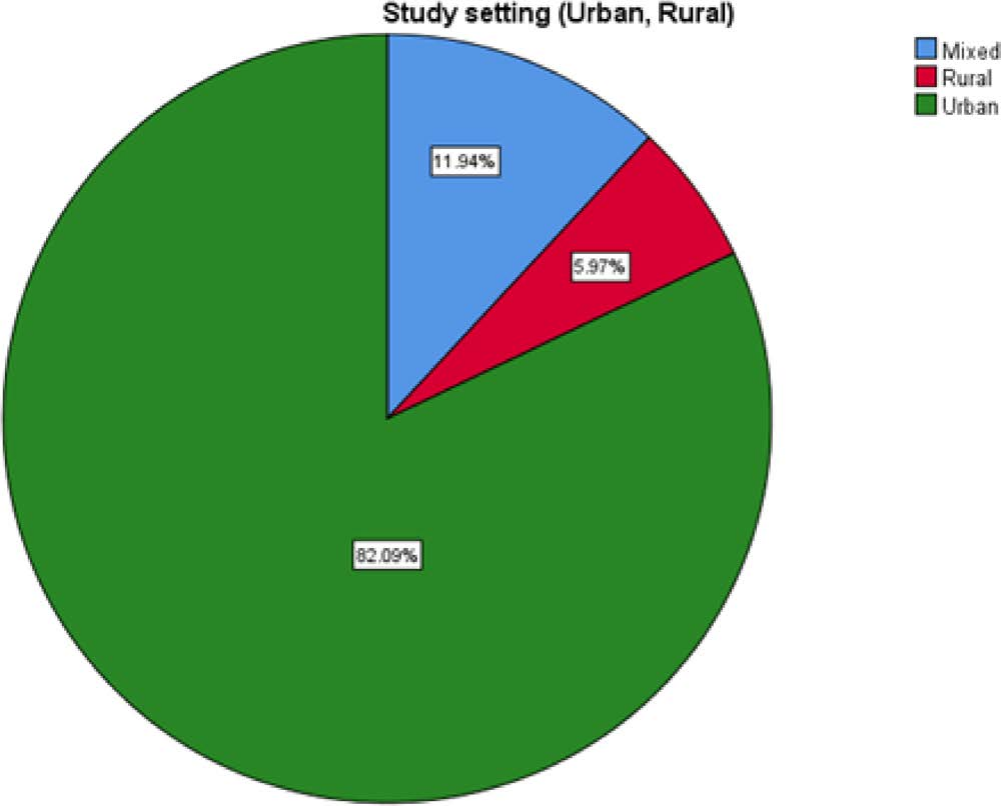
Pie chart depicting study setting (rural vs urban).

**Figure 8.**
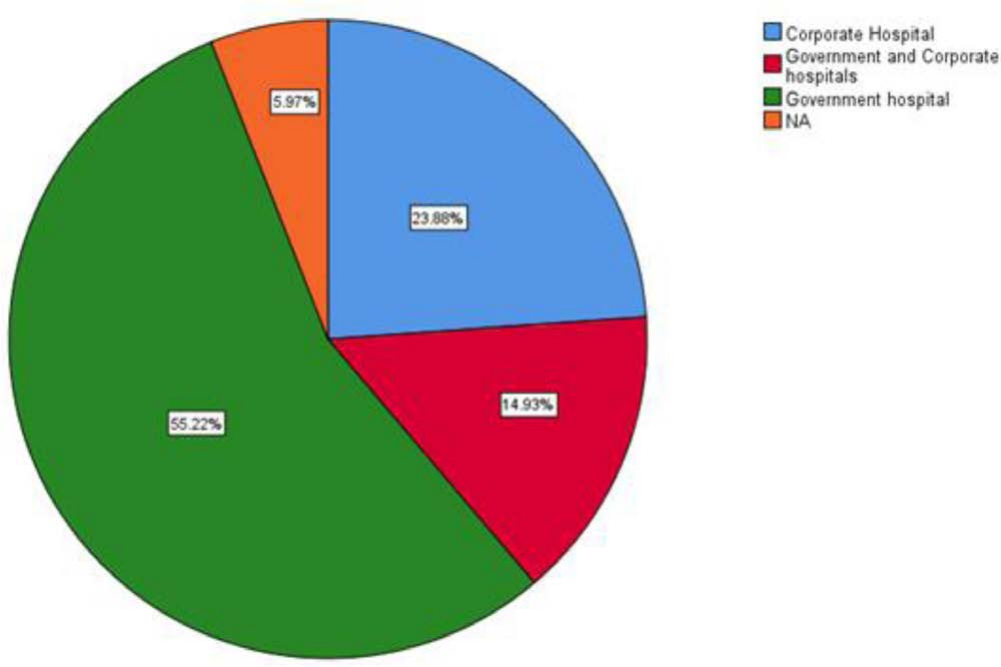
Pie chart depicting hospital types.

Data on study environment (urban, rural or mixed), hospital ownership and presence of a hospital stroke protocol were obtained from 67, 63 and 55 studies respectively. Eighty-two-point one percentage of studies were conducted in an urban setting while 11.9% were in a mixed urban and rural setting and the rest (6%) being in a rural setting (Fig. 7). Most of the studies were done in a government hospital (55.2%) with approximately a quarter of studies (25%) in a corporate hospital and 15% in a combined government and corporate hospital (Fig. 8). More than half of the hospitals had their own stroke protocol (52.2%). Lastly, among all the countries (n = 67) studied, less than half of them (40%) had a national stroke guideline.

### 3.2. Meta-analysis of rate of thrombolysis

**Figure 9.**
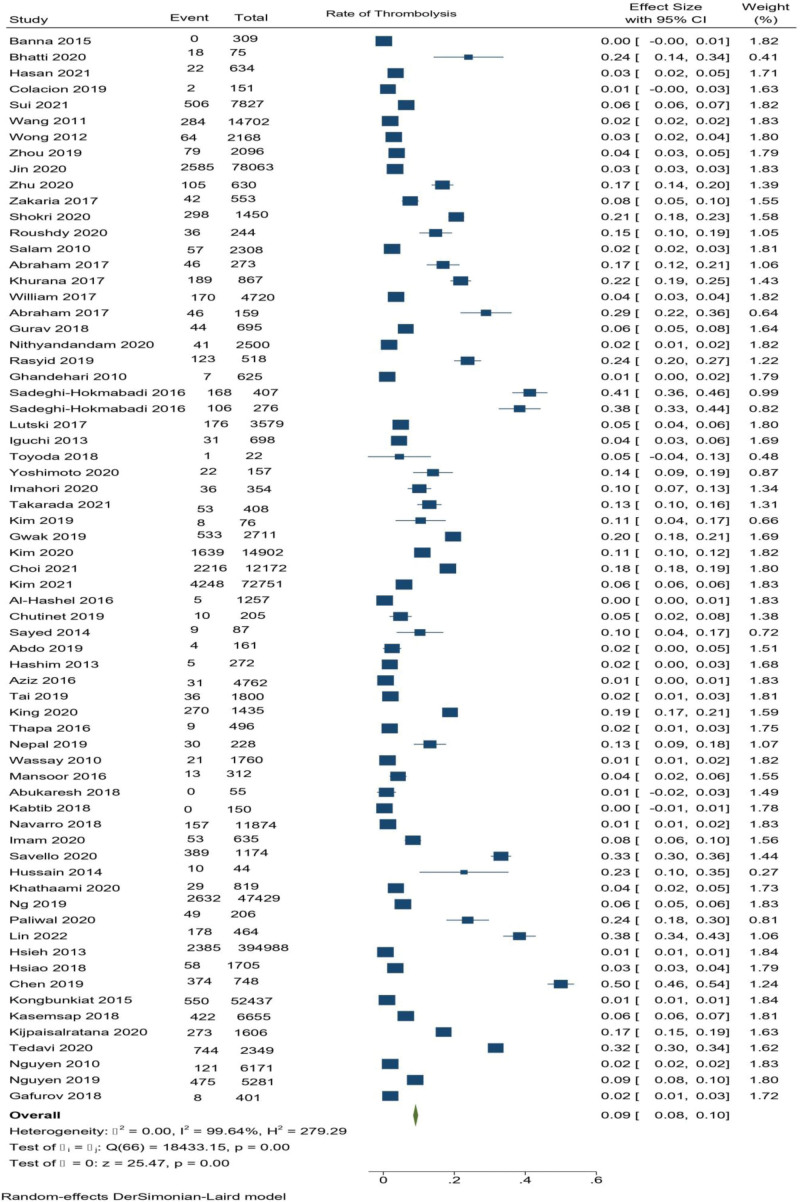
Forest plot with 95% CI showing pooled proportion of IV thrombolysis in Asian region. The area of each square is proportional to the study’s weight in the meta-analysis, while the diamond shows the pooled result. The horizontal lines through the square illustrate the length of the confidence interval. The width of the diamond serves the same purpose. The overall meta-analyzed measure of effect is an imaginary vertical line passing through the diamond. CI = confidence interval.

**Figure 10.**
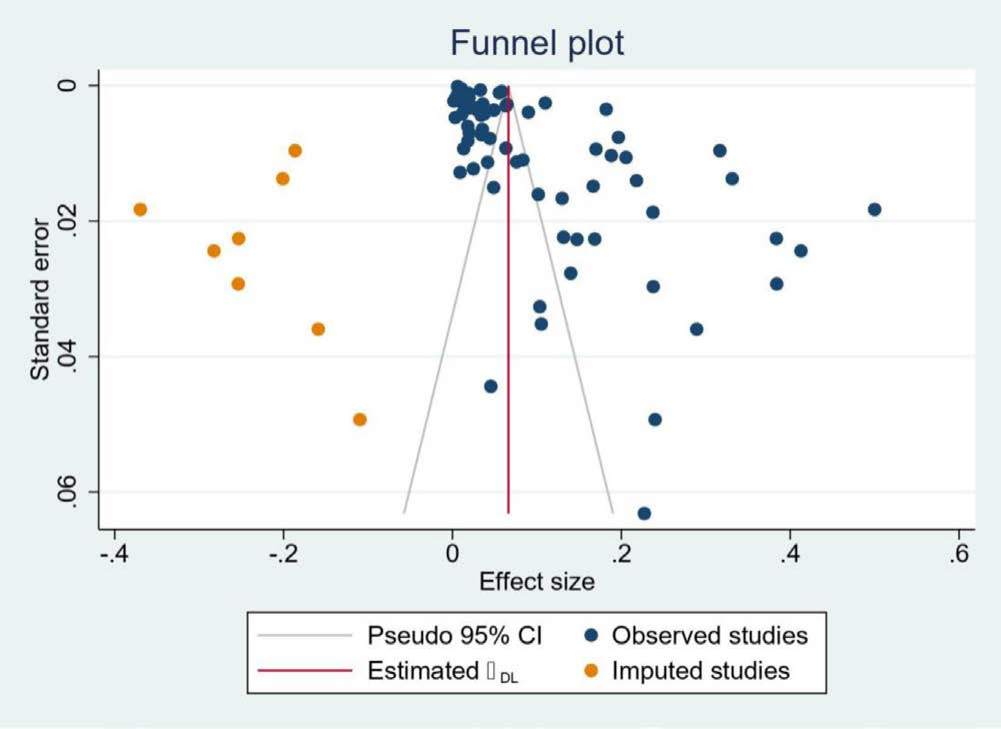
The funnel plot to assess the potential publication bias. Publication bias was observed and the adjusted proportion was estimated using the Duval and Tweedie trim and fill method (6.6%; 95% CI: 5.9%–7.3%; 8 studies imputed). CI = confidence interval.

The pooled proportion analysis of the 67 studies of Asia showed a rate of IV thrombolysis as 9.1% (95% CI 8.4%–9.8%, *P* < .001). Because of the high heterogeneity in our analysis (*I*^2^ = 99.64%, *P* < .001), we opted for Random effect model (DerSimonian-Laird model). The forest plot of the pooled proportion is illustrated in Figure 9. The funnel plot and Eggers test were carried out to assess the potential publication bias. Publication bias was observed (*P* < .001 in Eggers test). The adjusted proportion using the Duval and Tweedie trim and fill method was 6.6% of patients (95% CI: 5.9%–7.3%, 8 studies imputed) (Fig. 10). This suggests that the actual thrombolysis utilization rate may be slightly lower than the estimate obtained from the main analysis.

### 3.3. Subgroup analysis

To address the heterogeneity, subgroup analysis was conducted. The subgroup analysis was conducted on several headings: country of the study, Gross Domestic Product of the country, regions of the Asia, year of publication (before 2015 and 2015 onwards), study site, study setting, and hospital stroke protocol and stroke guideline. Results of subgroup analysis are illustrated in table 1.

**Table 1:** Subgroup variations in outcomes according to different predetermined factors.

Subgroups	No. of studies	Effect size	Lower CI	Upper CI	*I* ^2^	Subgroup difference
Country						
Bahrain	2	0.116	−0.118	0.349	95.71%	<0.001
Bangladesh	1	0.035	0.020	0.049	NA	
Brunei	1	0.013	−0.005	0.031	NA	
China	6	0.049	0.036	0.061	98.57%	
Egypt	3	0.143	0.052	0.234	97.15%	
India	7	0.097	0.070	0.124	98.12%	
Indonesia	1	0.237	0.201	0.274	NA	
Iran	3	0.269	−0.042	0.579	99.51%	
Israel	1	0.049	0.042	0.056	NA	
Japan	5	0.094	0.048	0.139	88.35%	
Korea	5	0.132	0.076	0.187	99.77%	
Kuwait	1	0.004	0.000	0.007	NA	
Laos	1	0.049	0.019	0.078	NA	
Lebanon	2	0.058	−0.018	0.135	80.31%	
Malaysia	4	0.057	0.018	0.095	99.05%	
Nepal	2	0.073	−0.038	0.184	95.83%	
Pakistan	2	0.025	−0.004	0.054	84.77%	
Palestine	2	0.004	−0.005	0.013	0%	
Philippines	1	0.013	0.011	0.015	NA	
Qatar	1	0.083	0.062	0.105	NA	
Russia	1	0.331	0.304	0.358	NA	
Saudi Arabia	2	0.121	−0.066	0.308	89.04%	
Singapore	2	0.144	−0.034	0.323	97.35%	
Taiwan	4	0.227	0.131	0.322	99.71%	
Thailand	3	0.080	0.024	0.137	99.66%	
Turkey	1	0.317	0.298	0.336	NA	
Uzbekistan	1	0.020	0.006	0.034	NA	
Vietnam	2	0.055	−0.014	0.124	99.62%	
Economy						
HIC	24	0.113	0.094	0.132	99.81%	0.013
LMIC	27	0.081	0.070	0.091	98.69%	
UMIC	16	0.090	0.075	0.105	99.44%	
Region						
Arab	18	0.124	0.088	0.160	99.22%	<0.001
East Asia	20	0.112	0.096	0.129	99.84%	
South Asia	12	0.069	0.052	0.086	97.12%	
South East Asia	15	0.068	0.054	0.081	99.53%	
Central Asia and North Asia	2	0.175	−0.130	0.481	99.76%	
Year of publication						
Before 2015	12	0.015	0.012	0.019	97.06%	<0.001
2015 onwards	55	0.113	0.103	0.124	99.45%	
Study site						
Urban	55	0.101	0.091	0.110	99.43%	0.045
Rural	4	0.109	0.023	0.195	98.65%	
Mixed	8	0.065	0.039	0.092	99.88%	
Study setting						
Government hospital	37	0.099	0.088	0.109	99.20%	<0.001
Corporate hospital	16	0.076	0.057	0.096	98.34%	
Government and corporate hospital	10	0.138	0.118	0.157	99.91%	
Hospital stroke protocol						
Present	34	0.107	0.096	0.117	99.61%	0.001
Absent	20	0.076	0.063	0.089	98.91%	
National stroke guideline						
Present	40	0.101	0.091	0.112	99.71%	0.064
Absent	27	0.085	0.071	0.099	99.39%	

CI = confidence interval, HIC = higher income countries, UMIC = upper-middle-income country.

Subgroup analysis by country showed that Russia has the highest rate of thrombolysis compared to other countries (n = 1, pooled proportion 33.1%, 95% CI 30.4%–35.8%). When differentiated using GDP of the country, pooled proportion of rate of thrombolysis was as follows: 11.3% (95% CI 9.4%–13.2%, n = 24 studies) in higher income countries, 8.1% (95% CI 7%–9.1%, n = 27 studies) in LMICs and 9% (95% CI 7.5%–10.5%, n = 16 studies) in upper-middle-income countries. When segregated by regions where studies were from, the highest rate of thrombolysis was observed in Central and North Asia (pooled proportion 17.5%, 95% CI −13% to 48%) while the lowest rate of thrombolysis was seen in Southeast Asia (pooled proportion 6.8%, 95% CI 5.4%–8.1%). Subgroup analysis by year of publication showed that studies conducted on or after 2015 has a higher rate of thrombolysis compared to studies conducted before 2015 (n = 55, pooled proportion 11.3%, 95% CI 10.3%–12.4% vs n = 12, pooled proportion 1.5%, 95% CI 1.2%–1.9%). In terms of hospital location, both urban and rural settings had similar rate of thrombolysis (n = 55, 10.1%, 95% CI 9.1%–11% for urban setting, n = 4, 10.9%, 95% CI 2.3%–19.5% for rural setting). In terms of hospital, results of meta-analysis showed that studies with data from both government and corporate hospital had the highest rate of thrombolysis (n = 10, pooled proportion 13.8%, 95% CI 11.8%–15.7%) than government hospital (n = 27, pooled proportion 9.9%, 95% CI 8.8%–10.9%) and corporate hospital (n = 16, pooled proportion 7.6%, 95% CI 5.7%–9.6%). Finally, presence of hospital stroke protocol and a national stroke guideline were associated with higher rates of thrombolysis among ischemic stroke patients (n = 34, pooled proportion 10.7%, 95% CI 9.6%–11.7% and n = 40, pooled proportion 10.1%, 95% CI 9.1%–11.2% respectively).

In summary, the subgroup difference was significant for country of study (*P* < .001), Regions of Asia (*P* = .026), Year of publication (*P* < .001), GDP (*P* = .013), study site (*P* = .045), study setting (*P* < .001) and presence of hospital stroke protocol (*P* = .001) while except for presence of national stroke guideline (*P* = .064).

## 4. Discussion

Home to almost half of the world’s population, Asia also has highest stroke burden in the world. Further, with high burden of chronic diseases like hypertension and diabetes, the incidence of stroke is on the rise with estimates between 116 and 483/100 000 per year. The highest burden is in countries like China and India and lowest in countries like Korea and Singapore. IVT and MT are proven treatment for AIS and improve clinical outcomes. However, the rate of IVT remains universally low-and more so in Asia.^[1,8–10]^

Our review found variability in the rate of thrombolysis across Asia with 11.3% receiving thrombolysis in HIC countries as opposed to only 8.1% in LMICs; way below the targeted rate of 50% set by American Stroke Association. This can be accounted for by prehospital delay, in hospital delay, lack of infrastructure and poor policy/governance surrounding stroke care.

The higher rate of thrombolysis in HIC could be explained by several factors including the available infrastructure and human resources. The number of neurologists varies from 68 in HICs like Japan, 35 in Taiwan, 26 in China, 22 in South Korea to <1 per million in Nepal, Pakistan, India and other LMICs. These discrepancies also exist in terms of number of CT scanners, MRI scanners, dedicated stroke units/beds, number of center capable of IVT and existence of national stroke guidelines.^[9–11]^ Further accessibility and high cost of standard IVT (alteplase) is a barrier in LMICs and deny many patients opportunities for IVT especially with lack of insurance systems. Alteplase, the most common thrombolytic used is quite expensive and its cost has doubled in the past decade. Patients in HIC are more likely to easily afford this costly treatment. Having combined public and private ownership of the hospital ensures availability of thrombolytic drugs as well as subsidy in its cost. However, there are low cost but equally efficacious alternatives including low dose alteplase, tenecteplase and should be encouraged to increase IVT rates across Asia.^[12,13]^

Apart from organizational and cost barriers, prehospital delay is one of the most significant contributors to low thrombolysis rates as IVT is permissible only within a window of 4.5 hours from the onset of symptoms. Failure to recognize stroke symptoms by patients/caregivers/physicians and lack of awareness of thrombolysis as a treatment was evident in our review and remains consistent factors across all studies in Asia.^[14–17]^ Further absence of witness at onset, improving symptoms after onset is other contributory factors.^[18]^ Prehospital care including ambulance services with paramedics trained to identify stroke symptoms/IVT, helicopter emergency medical service (HEMS) and lack of proper referral strategy paly role in prehospital delay.

Intra hospital delay or increased door to needle time is another important factor for low rates of IVT. Hospitals having their own stroke protocol and countries having national stroke guidelines have higher thrombolysis rates. Get with the guidelines- stroke, a randomized national quality improvement program focused on improving organizational elements increased the rate of thrombolysis by 30% in participating hospitals over the course of 4 years.^[19]^ Recently, the Helsinki model have shown to significant cut intrahospital delay. The model included ambulance prenotification with patient details alerting the stroke team to meet the patient on arrival; patients transferred directly from triage onto the CT table on the ambulance stretcher; and tissue plasminogen activator delivered in CT immediately after imaging. In studies conducted in Melbourne and Christchurch after adoption of Helsinki model, the team managed to reduce door to needle time to 25 and 34 minutes respectively.^[20,21]^ In prehospital Acute Stroke Triage protocol, Quain et al found increase in rate of thrombolysis by 16.7% by making a prehospital stroke assessment tool for ambulance officers, an ambulance protocol for hospital bypass for potentially thrombolysis-eligible patients, and prehospital notification of the acute stroke team.^[22]^ In the TeleMedical Project for integrative Stroke Care, Müller-Barna et al^[23]^ similarly found an increase in thrombolytic rate of 15.1% by using round the clock telemedicine service for rural hospitals.

The future of stroke care in Asia looks promising as evident by the fact that the number of neurologists and rates of thrombolysis have increasing trend. Subgroup analysis by year of publication showed that studies conducted on or after 2015 has a higher rate of thrombolysis compared to studies conducted before 2015. However, there is much work to be done to approach the rates seen in the Western world. Amongst them, the most notable ones are use of EMS/HEMS services including training paramedical staff,^[24]^ educating public and establishment of telestroke and mobile stroke units. In our review only 6 studies reported using EMS services, 4 reported using teleconsultation and 1 reported using both EMS and Teleconsultation services. HASTA study, a Stockholm based randomized control study, found that simply increasing EMS priority level of stroke patients to Level 1 (immediate call of an ambulance) from Level 2 (within 30 minutes ambulance) resulted in an absolute 14% increase in thrombolysis rate.^[25]^ Establishment of telestroke network using smart phones can help bridge the gap further and increase thrombolysis in resource poor settings.^[9,26,27]^ Effectiveness of public education in symptom recognition and promptly seeking health services was studied by Bray JE et al^[28]^ in Melbourne and found that Ambulance dispatches for stroke significantly increased after introducing the National Stroke Foundation public health education campaigns.

This is the largest review studying the thrombolysis trends across Asian countries which will inform the policy makers about the changing landscape of stroke care encouraging them to improve their policy. This is the biggest strength of the study. However, there are few limitations. First, majority of the studies included in our study were urban centric (up to 90%). This fails to capture all the stroke cases that never became eligible for IVT as stroke services were unavailable at local center or could not reach urban stroke center due to costly EMS/HEMS. Second, there is high heterogeneity among the individual studies. Several factors might have contributed to this heterogeneity, including socioeconomic disparities, variations in healthcare infrastructure and resources, lack of public awareness and education about stroke symptoms, presence of national stroke guidelines and hospital stroke protocols, prehospital and in hospital delays, and access to emergency medical services. Third, due to language barrier and possibly lack of research, few studies with IVT in ischemic stroke exist in the literature from Asia. Furthermore, out tight inclusion criteria might also have resulted in limited studies. This could under/overestimate the true estimates for IVT rates and may not always reflect real world scenario.

## 5. Conclusion

Patients with ischemic stroke in HIC countries, countries/hospitals with their own guidelines/protocols, living in urban areas, and receiving care in hospitals with a public-private partnership are more likely to receive thrombolysis, according to our findings. Improving thrombolysis rates throughout Asia would be greatly aided by reducing prehospital and hospital delays, enhancing local infrastructure and human resources, and educating the public about stroke.

## Author contributions

**Conceptualization:** Bikram Prasad Gajurel, Gaurav Nepal, Song Peng Ang, Sujan Bohara, Sanjeev Kharel, Jayant Kumar Yadav.

**Data curation:** Bikram Prasad Gajurel, Gaurav Nepal, Sanjeev Kharel.

**Formal analysis:** Bikram Prasad Gajurel, Nishat Shama, Jayant Kumar Yadav.

**Investigation:** Gaurav Nepal, Song Peng Ang, Sujan Bohara, Sanjeev Kharel, Jayant Kumar Yadav.

**Methodology:** Gaurav Nepal, Song Peng Ang, Priyanshu Nain, FNU Ruchika, Jayant Kumar Yadav.

**Project administration:** Gaurav Nepal, Vikash Jaiswal, Song Peng Ang, Priyanshu Nain, Nishat Shama, Sujan Bohara, FNU Ruchika, Sanjeev Kharel.

**Resources:** Jayant Kumar Yadav.

**Software:** Gaurav Nepal, Vikash Jaiswal, Nishat Shama.

**Supervision:** Gaurav Nepal, Vikash Jaiswal, Jayant Kumar Yadav.

**Validation:** Gaurav Nepal, Vikash Jaiswal, Priyanshu Nain, Sujan Bohara, Sanjeev Kharel.

**Visualization:** Gaurav Nepal.

**Writing – original draft:** FNU Ruchika, Jillian Reeze T. Medina, Abhigan Babu Shrestha.

**Writing – review & editing:** FNU Ruchika, Jillian Reeze T. Medina, Abhigan Babu Shrestha.

## Supplementary Material




