# Supplementary material for: Utilization rates of intravenous thrombolysis for acute ischemic stroke in Asian countries:: A systematic review and meta-analysis
Source: Medicine (Baltimore). 2023 Oct 20;102(42):e35560. doi: 10.1097/MD.0000000000035560 (PMC10589571; doi:10.1097/MD.0000000000035560)
Supplement: Supplementary file 1 [file medi-102-e35560-s001.docx]

**Supporting information: Search Strategy**

**Date of Search**: 7th April, 2022

**Filters Used:**  English

**Search syntax:** (“Term_m1” OR “Term_m2” OR “Term_m3”...) AND (“Term_n1” OR “Term_n2” OR “Term_n3”...) AND ( ‘cross-sectional study’ OR ‘prevalence’ OR

‘rate’) AND (“Name of Country”)

**“Term_m”s used were:**

1. Cerebrovascular Accident
2. Ischemic stroke [MeSH Terms]
3. Brain Infarction
4. Cerebral Infarction

**“Term_n”s used were:**

1. Thrombolytic Therapy [Mesh]
2. Tissue Plasminogen Activator [Mesh]
3. Alteplase
4. Urokinase
5. Tenecteplase
6. Thrombolysis
7. rt-PA

**Names of Countries and Cities Used were:**

1. Afghanistan
2. Armenia
3. Azerbaijan
4. Bahrain
5. Bangladesh
6. Bhutan
7. Brunei
8. Myanmar
9. Cambodia
10. China
11. Egypt
12. Georgia
13. Hong Kong
14. India
15. Indonesia
16. Iran
17. Iraq
18. Israel
19. Japan
20. Jordan
21. Kazakhstan
22. North Korea
23. South Korea
24. Kuwait
25. Kyrgyzstan
26. Laos
27. Lebanon
28. Macau
29. Malaysia
30. Maldives
31. Mongolia
32. Nepal
33. Oman
34. Pakistan
35. Philippines
36. Qatar
37. Russia
38. Saudi Arabia
39. Singapore
40. Sri Lanka
41. Syria
42. Taiwan
43. Tajikistan
44. Thailand
45. Timor-Leste
46. Turkmenistan
47. Turkey
48. United Arab Emirates
49. Uzbekistan
50. Vietnam
51. Yemen

Time frame: 2010 to 2021(December), English, Human subjects

**PUBMED**: 259

((("Ischemic Stroke"[MeSH Terms] OR "Cerebral Infarction" OR "Brain Infarction") AND ("Thrombolytic Therapy"[Mesh] OR "Tissue Plasminogen Activator"[Mesh] OR "alteplase" OR "urokinase" OR "Tenecteplase" OR "thrombolysis" OR "rt-PA")) AND ("cross-sectional study" OR "prevalence" OR "rate")) AND ("Afghanistan" OR "Armenia" OR "Azerbaijan" OR "Bahrain" OR "Bangladesh" OR "Bhutan" OR "Brunei" OR "Myanmar" OR "Cambodia" OR "China" OR "Egypt" OR "Georgia" OR "Hong Kong" OR "India" OR "Indonesia" OR "Iran" OR "Iraq" OR "Israel" OR "Japan" OR "Jordan" OR "Kazakhstan" OR "North Korea" OR "South Korea" OR "Kuwait" OR "Kyrgyzstan" OR "Laos" OR "Lebanon" OR "Macau" OR "Malaysia" OR "Maldives" OR "Mongolia" OR "Nepal" OR "Oman" OR "Pakistan" OR "Philippines" OR "Qatar" OR "Russia" OR "Saudi Arabia" OR "Singapore" OR "Sri Lanka" OR "Syria" OR "Taiwan" OR "Tajikistan" OR "Thailand" OR "Timor-Leste" OR "Turkmenistan" OR "Turkey" OR "United Arab Emirates" OR "Uzbekistan" OR "Vietnam" OR "Yemen")
