# Supplementary figures and images for: Utilization rates of intravenous thrombolysis for acute ischemic stroke in Asian countries:: A systematic review and meta-analysis
Source: Medicine (Baltimore). 2023 Oct 20;102(42):e35560. doi: 10.1097/MD.0000000000035560 (PMC10589571; doi:10.1097/MD.0000000000035560)

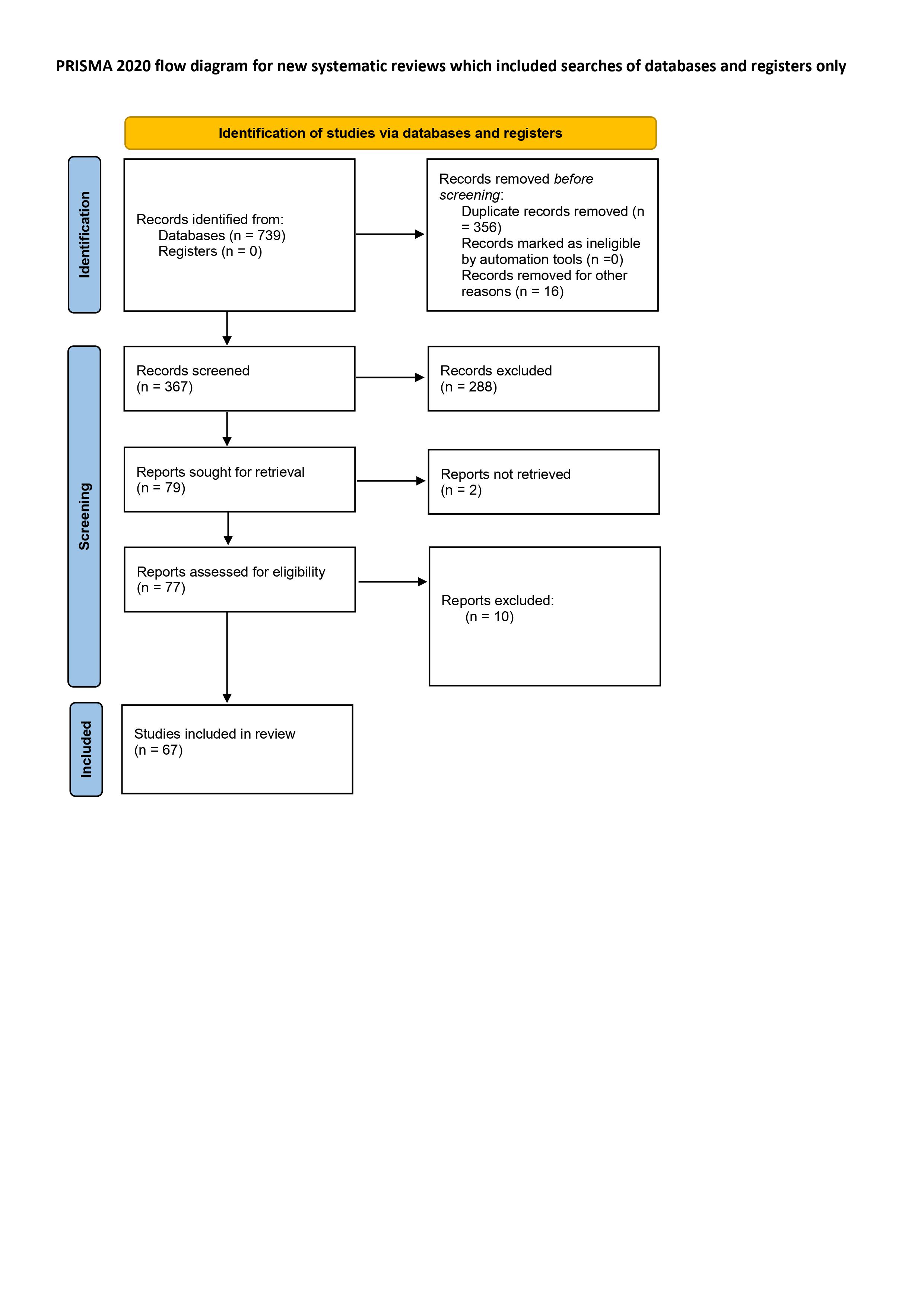

Supplement: Supplementary file 2 [file medi-102-e35560-s002.jpg]
